# Spt5′s central KOW domains and the Pol II stalk collaborate to regulate chromatin and 3′-end processing in *Saccharomyces cerevisiae*

**DOI:** 10.1093/g3journal/jkag123

**Published:** 2026-05-11

**Authors:** Zachary A Morton, Michael J Doody, Nayeli Naik, Nancy Paniagua, Claire Delahunty, John R Yates, Carlos Bustamante, Grant A Hartzog

**Affiliations:** Department of Molecular, Cell and Developmental Biology, University of California, Santa Cruz, Santa Cruz, CA 95064, United States; California Institute for Quantitative Biosciences, University of California, Berkeley, CA 94720, United States; Department of Molecular, Cell and Developmental Biology, University of California, Santa Cruz, Santa Cruz, CA 95064, United States; Department of Molecular, Cell and Developmental Biology, University of California, Santa Cruz, Santa Cruz, CA 95064, United States; Department of Molecular, Cell and Developmental Biology, University of California, Santa Cruz, Santa Cruz, CA 95064, United States; Department of Biochemistry, Stanford University School of Medicine, Stanford, CA 94305, United States; Department of Integrative Structural & Computational Biology, The Scripps Research Institute, La Jolla, CA 92037, United States; Department of Integrative Structural & Computational Biology, The Scripps Research Institute, La Jolla, CA 92037, United States; California Institute for Quantitative Biosciences, University of California, Berkeley, CA 94720, United States; Howard Hughes Medical Institute, University of California, Berkeley, CA 94720, United States; Jason Choy Laboratory of Single Molecule Biophysics, University of California, Berkeley, CA 94720, United States; Biophysics Graduate Group, University of California, Berkeley, CA 94720, United States; Department of Chemistry, University of California, Berkeley, CA 94720, United States; Kavli Energy Nanoscience Institute, University of California, Berkeley, CA 94720, United States; Department of Physics, University of California, Berkeley, CA 94720, United States; Molecular Biophysics and Integrative Bioimaging Division, Lawrence Berkeley National Laboratory, Berkeley, CA 94720, United States; Department of Molecular and Cell Biology, University of California, Berkeley, CA 94720, United States; Department of Molecular, Cell and Developmental Biology, University of California, Santa Cruz, Santa Cruz, CA 95064, United States

**Keywords:** Spt5, stalk, Rpb4, Rpb7, termination, Nrd1, transcription, chromatin

## Abstract

Spt5 is a universally conserved multidomain transcription elongation factor that acts as a component of all Pol II elongation complexes (ECs). Structural studies indicate that several of Spt5′s central KOW domains lie adjacent to the Pol II stalk, composed of subunits Rpb4 and Rpb7. However, the *in vivo* functions of Spt5′s central KOW domains are unknown. Here, we show that Spt5 and Rpb4/7 jointly modulate 3′-end formation and co-transcriptional chromatin integrity in *Saccharomyces cerevisiae*. We identify mutations in the *SPT5 KOW2-3* domains and *RPB7* that cause cryptic initiation of transcription and alter 3′-end formation of RNA transcripts. Molecular readthrough assays reveal allele-specific changes at both *GAL10* and *SNR13*, consistent with impacts on termination mediated by Cleavage and Polyadenylation Factor (CPF)–Cleavage Factor (CF) and the Nrd1–Nab3–Sen1 (NNS) pathway. Proteomic experiments with isolated KOW domains enrich factors from both pathways as well as chromatin regulators, overlapping known Rpb7 interactors. Together, these findings support a model in which the Spt5 KOW2-4/Pol II stalk region acts as a recruitment platform that coordinates pre-mRNA processing and chromatin dynamics during elongation, revealing new roles for the central KOW domains of Spt5.

## Introduction

Spt5 is an essential, multidomain transcription elongation factor conserved across bacteria, archaea, and eukaryotes. In archaea and eukaryotes, Spt5 has a conserved binding partner in Spt4, a small zinc-finger protein that stabilizes Spt5 (Malone et al. 1993; Ding et al. 2010). The bacterial homolog of Spt5, NusG, is composed of an N-terminal NGN domain and a C-terminal KOW domain. NusG's NGN domain promotes transcription processivity by sealing DNA in the RNA polymerase central cleft and clamping RNA polymerase on DNA (Wang and Artsimovitch 2020). This function is conserved in eukaryotes. While NusG contains only a single KOW domain, eukaryotes have up to 7 (5 in *S. cerevisiae,* 4 to 7 in metazoans) (Pandey et al. 2023). While the conserved role of the NGN domain has been well-defined, the precise functions of Spt5′s central KOW domains remain ambiguous. As the expansion of Spt5′s KOW domains is eukaryote specific, we hypothesize that their function may be related to eukaryote specific transcriptional challenges, such as navigating chromatin or pre-mRNA processing. Indeed, in yeast, Spt5 is known to facilitate splicing, mRNA export, transcription through chromatin and chromatin maintenance (Lindstrom et al. 2003; Mayer et al. 2012; Hartzog and Fu 2013; Crickard et al. 2017; Evrin et al. 2022; Song and Chen 2022). Supporting this model, structural studies have placed several KOW domains adjacent to the Pol II stalk near the mRNA exit channel, far from the catalytic core of Pol II (Bernecky et al. 2017; Ehara et al. 2017).

Rpb4 and Rpb7 are Pol II subunits that comprise the stalk region of Pol II in eukaryotes. These Pol II subunits are unique in that they can reversibly dissociate from the body of Pol II and are sub-stoichiometric to the remainder of Pol II (Tan et al. 2003). Rpb4/7 are involved in every step in transcription, from initiation to termination (Kolodziej et al. 1990; Edwards et al. 1991; Choder and Young 1993; Choder 2004; Sharma and Kumari 2013; Allepuz-Fuster et al. 2019; Calvo 2020) and have been shown to have roles in recruitment of 3′-end processing factors and polyadenylation site choice (Runner et al. 2008; Moqtaderi et al. 2026). Additionally, Rpb7 has been shown to bind nascent mRNA (Meka et al. 2005; Ujvári and Luse 2006). Structurally, the Pol II stalk protrudes from the foot domain of Pol II, and Rpb7 serves to lock Pol II into a more processive conformation (Armache et al. 2005). The exposed position of Rpb4/7 away from the body of Pol II suggests it can attract diverse functional units to the transcription complex (Runner et al. 2008; Sharma and Kumari 2013; Schier and Taatjes 2020). Because KOW2-4 lie alongside Rpb4/7 near the mRNA exit channel (Bernecky et al. 2017; Ehara et al. 2017; Qiu and Gilmour 2017), the combined KOW-stalk surface is well placed to impact termination pathway selection and messenger ribonucleoprotein (mRNP) maturation.

The positioning of Spt5′s central KOW domains adjacent the Pol II stalk suggests that their functions may be functionally coordinated. Indeed, both Spt5 and Rpb4/7 have been shown to contribute to transcription elongation processivity (Armache et al. 2003; Li et al. 2014; Bernecky et al. 2017; Ehara et al. 2017), mRNA export (Farago et al. 2003; Lindstrom et al. 2003; Lotan et al. 2005; Harel-Sharvit et al. 2010), recruitment of 3′ processing factors and poly(A) site choice (Cui and Denis 2003; Mitsuzawa et al. 2003; Runner et al. 2008; Mayer et al. 2012), as well as repression of transcription-coupled repair (TCR) (Jansen et al. 2000; Li and Smerdon 2002; Ding et al. 2010; Li et al. 2014; Gong and Li 2023). Notably, recent work has shown that contact between Spt5-KOW3 and Rpb7 is required for proper repression of Rad26-mediated TCR, providing evidence for a functional interplay between Spt5 and the Pol II stalk in transcription-associated processes (Gong and Li 2023). These observations motivate a model in which Spt5 and the Pol II stalk function together to regulate multiple aspects of transcription elongation, which we test in this study.

Here we test the hypothesis that the Spt5 KOW2-4/Pol II stalk region modulates co-transcriptional chromatin structure and 3′-end formation in yeast. We combine allele-specific genetics, molecular readthrough assays at *GAL10* and *SNR13*, and KOW-domain pulldown proteomics. We find that *spt5* KOW2-3 and *rpb7* alleles differentially affect cryptic initiation and polyadenylation (poly(A)) site choice as well as modulate CPF/CF and NNS mediated termination. Further, Spt5 KOW2-3 and KOW4 proteomics reveal enrichment for chromatin-related factors as well as factors involved in both canonical yeast termination pathways (CPF/CF and NNS), which overlap with known Rpb7 interactors, supporting a model in which the KOW-stalk surface serves as a context-dependent recruitment platform during elongation.

## Materials and methods

### Media and yeast genetic methods

Yeast media and genetic manipulations followed standard methods (Rose et al. 1990). Complete strain genotypes are listed in Supplementary Table 1 (isogenic to S288C; *GAL2+*). Strain GHY1555 (originally CKY297 from the Kaplan lab) was a gift of Craig Kaplan and FY603 was a gift of Fred Winston. All other strains are from the Hartzog lab collection.

GHY4006 and GHY4007 were generated via a marker switch of GHY3349 and GHY3348, respectively harboring CKB310. PCR amplification of pRS404 was performed using OGH473 and OGH474 (1 × Mango Mix (Bioline), 30 cycles of 95 °C 30 s, 51 °C 1 min, 72 °C 90 s). High efficiency transformation was performed and cells were plated onto –Trp to select for plasmids in which *TRP1* replaced *LEU2.* Transformants were then replica plated to –Trp –Leu to screen for colonies that failed to grow, indicating loss of the *LEU2* marker on CKB310.

All single-mutant analyses in *SPT5* and *RPB7* were performed via a plasmid shuffle approach. The indicated strains were transformed with *spt5  LEU2* or *rpb7  LEU2* centromeric plasmids, selected via growth on SC–Leu, and replica plated to 5-FOA for counterselection prior to phenotypic analysis. Double mutant analyses were performed similarly, except transformants were selected from SC–Leu, grown in YPD, and spotted directly onto indicated 5-FOA media. All molecular analyses were performed on strains that have been passaged over 5-FOA.

### Plasmids

A detailed list of the plasmids used in this study is provided in Supplementary Table 2.

Plasmid pZM6 was generated by PCR mutagenesis. Plasmid CKB223 was amplified with either OGH1630 + OGH1631 or OGH1629 + OGH1632 (Phusion polymerase, 1 × Phusion HF Buffer (Thermo Scientific), 0.1 mM dNTPs; 30 cycles 95 °C 2 min, 62 °C 1 min, 70 °C 30 s). Gel excision was performed on the products using NucleoSpin Gel and PCR cleanup kit (Macherey-Nagel). Five microliters of each product was added to a new reaction under the same conditions lacking primers and cycled 5 times (95 °C 30 s, 65 °C 1 min, 70 °C 45 s). OGH1631 and OGH1632 were then added and cycled 30×. CKB233 and the PCR product were individually digested with *Xho*I and *Eag*I and the digested PCR product was ligated into the CKB233 backbone.

To integrate *spt5-E546K* and *spt5-G587D*, a high efficiency transformation was performed with plasmids pMD11 and pMD18 that were digested with *Eag*I and *Hind*III into GHY3246 and GHY2741, respectively. Transformants were recovered after 24 h at 30 °C on YPD, replica plated to 5-FOA for counter-selection (2 × 48 h, 30 °C), then to 5-FOA –Trp. Stable Trp- colonies were restreaked and verified by PCR/Sanger sequencing.

### Oligonucleotides

A detailed list of oligonucleotides used in this study is provided in Supplementary Table 3. OGH1773, OGH1774, OGH1775, and OGH1776 sequences were originally used in Lee et al. 2020.

### Hydroxylamine mutagenesis-based screens

Plasmids were mutagenized with hydroxylamine as described (Rose et al. 1990). Briefly, 10 µg of plasmid DNA was incubated in 1 M hydroxylamine for 20 h at 37 °C. Reactions were quenched with 10 µL 5 M NaCl and 50 µL 1 mg/ml BSA and ethanol precipitated. Pellets were resuspended in TE, re-precipitated, pelleted, air-dried, and resuspended in 100 µL TE. Mutagenized DNA was used directly for transformation of *S. cerevisiae* by standard high-efficiency methods (GHY1555 for *RPB7  gal10Δ56,* GHY3244 for *RPB7* cryptic initiation, GHY2741 for *SPT5* cryptic initiation). Transformants were plated onto SC -Leu to select for transformants that contained the mutant *SPT5* or *RPB7* plasmids. Each strain was passed over 5-FOA twice to counter-select against the wild-type *URA3* plasmid. These plates were then replica plated to selective media (standard YP media with 2% galactose [YPGal] for *gal10Δ56,* SC -His with 2% galactose for cryptic initiation) and allowed to grow at 30 °C for 2 d to assay for the phenotype of interest. Colonies that grew on the selective media were re-streaked on YPD and then replica plated again to selective media and allowed to grow at 30 °C for 2 d to confirm the phenotype. SC -HIS + 2% glucose and YPGal were used as controls for cryptic initiation to ensure there was no leaky *HIS3* expression under noninducing conditions. Plasmids were rescued and transformed into DMSO competent DH5α *E. coli,* retransformed into *S. cerevisiae* to confirm the phenotype is plasmid-dependent, and then subjected to Sanger sequencing. Phenotypes were finally scored via serial dilution assay on selective media.

### Serial dilution assay

About 1 × 10^7^ cells from an overnight culture (30 °C, YPD) were resuspended in 1 mL of sterile water. The 5 5-fold serial dilutions were spotted onto agar plates containing the indicated media and allowed to grow for 48 h at 30 °C (or as specified).

### Spt5-KOW domain affinity chromatography

BL21-DE3 cells harboring plasmids pGH382 (K2K3-His6) and pGH378 (L2K4-His6) were inoculated from a single colony in 10 mL LB + kanamycin at 37 °C and grown overnight on a rotator. One milliliter of culture was used to inoculate 1.3 L of LB + at 37 °C and grown to OD 0.7 on an orbital shaker. Cultures were then induced with IPTG to 0.5 mM and shaken at 20 °C overnight, harvested, and frozen in liquid nitrogen. Frozen cell pellet was ground to a fine powder with a mortar and pestle frozen with liquid nitrogen and scooped into screw-cap tubes and frozen again in liquid nitrogen. Eight vials of cells were allowed to thaw on ice and mixed each with 0.5 mL of phosphate buffer (P-Buffer) (50 mM phosphate pH 7.8, 10% glycerol, 50 mM NaCl, 50 mM Arginine, 50 mM Glutamate, PMSF 1 × added fresh). Cells were then subjected to 3 rounds of sonication at 4 °C, each round being 30 s followed by 30 s on ice. Cells were spun for 10 min at 10,000 rpm in a microfuge at 4 °C. 1 mL HisPur Ni-NTA beads (Thermo Scientific) were equilibrated in P-Buffer, then placed in a chromatography column along with 4 mL of cell extract and placed on a rocker at 4 °C for 30 min. Column was washed with 20 CV P-Buffer at 250 mM NaCl, followed by 20 CV P-Buffer with 0.1% NP-40, followed by 20 CV P-Buffer with 100 mM NaCl and 4 mM imidazole. Column was eluted with 10,500 µL fractions of P-Buffer with 200 mM imidazole and frozen in liquid nitrogen. Fractions were then analyzed via SDS-PAGE. Five fractions containing the highest quantity of protein as determined by SDS-PAGE were buffer-exchanged and concentrated using Amicon Ultra-3 kDa MWCO centrifugal filters (3 exchanges; 4,000 rpm, 40 min each) into 25 mM MES. Purified protein (either K2K3, L2K4, or BSA) was incubated with Affi-Gel 10 resin with end-over-end mixing at 4 °C until the supernatant protein plateaued by Bradford (∼2.5 h). Reaction was quenched with 1 M ethanolamine for 1 h at 4 °C. One milliliter of Affi-gel from each of K2K3, L2K4, or BSA couplings were transferred to separate chromatography columns, drained and washed with 10 CV of yeast lysis buffer. About 25 mg crude yeast extract generated from GHY610 was diluted in 50 mL lysis buffer and loaded onto each column over a period of 8 h. Columns were washed with 10 CV yeast lysis buffer with 0.5% NP-40 at 0.2 M K(Ac), then 5 CV yeast lysis buffer without NP-40. 8 fractions of 400 µL each were eluted with a K(Ac) step-gradient (0.3–1.0 M in lysis buffer), starting with 0.3 M and ending in 1 M, followed by a 2.5 M urea strip. Fractions were split into 2 aliquots, frozen in liquid nitrogen, and stored at −80 °C. Half of each fraction was TCA precipitated and analyzed via SDS-PAGE on a silver-stained gradient gel. Fractions containing 0.8 M, 0.9 and 1 M K(Ac) containing ∼8–10 µg of protein were TCA precipitated and subjected to MudPIT mass spectrometry analysis.

### Yeast extract preparation

Mid-log phase cells were harvested via centrifugation and frozen in liquid nitrogen. Cell pellets were ground into a fine powder using a mortar and pestle under liquid nitrogen. Cell powder was allowed to thaw on ice and then mixed with an equal volume of yeast lysis buffer (for affinity chromatography: 30 mM HEPES, pH 7.4, 200 mM potassium acetate (K(Ac)), 1 mM magnesium acetate, 1 mM EGTA, 0.05% Tween-20, 10% glycerol. 1 mM PMSF, 2 µM Pepstatin A, 2 µg/mL Chymostatin, 0.6 µM Leupeptin, 2 mM Benzamidine HCl were added fresh. For co-IP: 6 mM Na_2_PO_4_, 4 mM NaH_2_PO_4_·H_2_O, 1% NP-40, 150 mM NaCl, 2 mM EDTA, 1 mM EGTA, 50 mM NaF, 4 µg/mL Leupeptin, 0.1 mM Na_2_VO_4_, 5% glycerol. To 50 mL of buffer, 1 complete protease inhibitor cocktail tablet (Roche) was added along with 130 µL 0.5 M benzamidine, 500 µL 100 mM PMSF). Cells were then centrifuged at 14,000 rpm for 10 min at 4 °C and supernatant collected. For affinity chromatography experiments, supernatant was clarified using an ultracentrifuge at 108,628 × *g* for 30 min at 4 °C.

### Mass spectrometry

Sample pellets were resuspended in 60 µL of buffer (8 M urea 100 mM Tris pH 8.5) and reduced with 3 µL of 100 mM Tris (2-carboxyethyl)phosphine hydrochloride (TCEP). Samples were alkylated in the dark for 20 min with 250 mM iodoacetamide and digested with trypsin overnight at 37 °C. Samples were acidified with formic acid (5%), and approximately 0.5 µg of sample was loaded onto EvoTips (Evosep) according to the manufacturer's protocol. Samples were run on an Evosep One (Evosep) coupled to a timsTOF Pro mass spectrometer (Bruker Daltonics). Peptides were separated with a gradient of buffer A (0.1% formic acid in H_2_O) and buffer B (0.1% formic acid in acetonitrile) on a 15 cm × 150 μm ID column with BEH 1.7 μm C18 beads (Waters) and an integrated tip pulled in-house. MS scans were acquired in PASEF mode, with 1 MS1 TIMS-MS survey scan and 10 PASEF MS/MS scans per 1.1 s acquisition cycle. Both ion accumulation time and ramp time in the dual TIMS analyzer were set to 100 ms, and the ion mobility range was 1/K0 = 0.6 to 1.6 V s cm^−2^. The m/z range was 100–1700. Precursor ions selected for MS/MS analysis were isolated with a 2 Th window for m/z < 700 and 3 Th window for m/z > 700. Collisional energy was lowered linearly from 59 eV at 1/K0 = 1.6 V s cm^−2^ to 20 eV at 1/K0 = 0.6 V s cm^−2^ as a function of increasing mobility. Precursors for MS/MS were picked at an intensity threshold of 2500, a target value of 20,000, and an active exclusion of 24 s. Singly charged precursor ions were excluded with a polygon filter. Tandem mass spectra were extracted from raw files using RawExtract (Version 1.9.9) and were searched using ProLuCID against a standard UniProt *Saccharomyces cerevisiae* database concatenated with reversed sequences. A static modification of carbamidomethylation on cysteine (57.02146) was considered. Data were searched with 50 ppm precursor ion tolerance and 600 ppm fragment ion tolerance. Data were filtered using DTASelect2 to a protein false-positive rate of <1%. A minimum of 2 peptides per protein and 1 tryptic end per peptide were required. Statistical models for peptide mass modification (modstat) and tryptic status (trypstat) were applied. Raw data have been deposited into the MassIVE repository (see data availability statement).

### RNA extraction and RT-qPCR analysis

Cells were grown to mid-logarithmic phase in YPD and 10 mL of cells were harvested for RNA extraction. For analysis of *GAL10* transcripts, cells were grown in YP Raffinose to mid-logarithmic phase and induced with 2% galactose for 1 h before harvest. RNA was extracted with phenol:chloroform:isoamyl alcohol (25:24:1) (PCI) at 65 °C for 10 min with intermittent vortexing. RNA was purified, precipitated, and resuspended in RNase free water. Genomic DNA was removed using DNase I (New England Biolabs), and RNA was PCI purified, precipitated, and resuspended in RNase free water. cDNA was prepared using combined random hexamer and oligo-dT primers (LunaScript RT SuperMix Kit, New England Biolabs) according to manufacturer instructions. qPCR amplification of cDNA was performed using Luna universal qPCR master mix according to manufacturer instructions using a Bio-Rad CFX96 Touch Real-Time PCR Detection System with melt-curve verification. Relative quantification used the ΔΔCt method. All primer sequences are detailed in Supplementary Table 3. For *GAL10*, qPCR signal for the readthrough product (OGH1664, OGH1665) was normalized to the *GAL10* ORF (OGH342, OGH343). For *SNR13* (OGH1673, OGH1674), qPCR signal was normalized to *ACT1* (OGH1675, OGH1676). 3 biological replicates were assessed for each genotype. For *GAL10* readthrough experiments, strains used were GHY1555, GHY3244, and GHY3368. For *SNR13* readthrough experiments, GHY3244, GHY3350, GHY4006, GHY4007, and GHY3368 were used. The indicated alleles of *RPB7* were transformed and the strain was passed over 5-FOA to counter-select for loss of the *RPB7  URA3* plasmid prior to analysis.

### Nrd1 Co-IP

Two milligrams of protein from crude yeast extract from FY603 (mock), GHY3244 + GHB464 (Rpb3-TAP), GHY3368 + GHB1417 (Rpb3-TAP, Spt5-E546 K + Rpb7-D166G) was incubated with 20 µL settled IgG Sepharose beads (Cytiva), preequilibrated in lysis buffer, and end-over-end rotated for 2 h at 4 °C. Beads were washed 3 times with TEV wash buffer (10 mM Tris-HCl, pH 8.0, 150 mM NaCl, 0.1% NP-40) and resuspended in 40 µL of TEV wash buffer with 1 mM DTT. On-bead TEV cleavage was performed by adding 2 µL of in-house purified TEV protease (200 mM stock) followed by end-over-end rotation overnight at 4° C. Supernatant was removed, and proteins were separated via 10% SDS-PAGE gel (acrylamide/bis) and transferred to nitrocellulose via wet transfer (100 V, 90 min, 4 °C). Membrane was blocked with 5% milk in TBS-T for 1 h, probed with rabbit anti-Nrd1 (1:5,000) followed by goat antirabbit HRP (1:5,000), stripped (0.2 M glycine, 0.1% SDS w/v, 1% Tween-20 v/v, pH 2.2) at RT 2 × 15 min, washed, re-blocked, and probed with goat anti-Rpb1 (1:2,000) followed by donkey antigoat HRP (1:5000). Images were collected using Bio-Rad Chemi Doc MP and analyzed using FIJI.

### Antibodies

Nrd1 antibody was generously provided by Dr. David Brow. Antirabbit was acquired from Invitrogen (31,460). Antigoat antibody was acquired from Invitrogen (A15999). Rpb1 antibody was acquired from Santa Cruz Biotechnology.

## Results

### Spt5 cryptic initiation alleles map adjacent to the Pol II stalk and suppress *gal10Δ56*

**Fig. 1.**
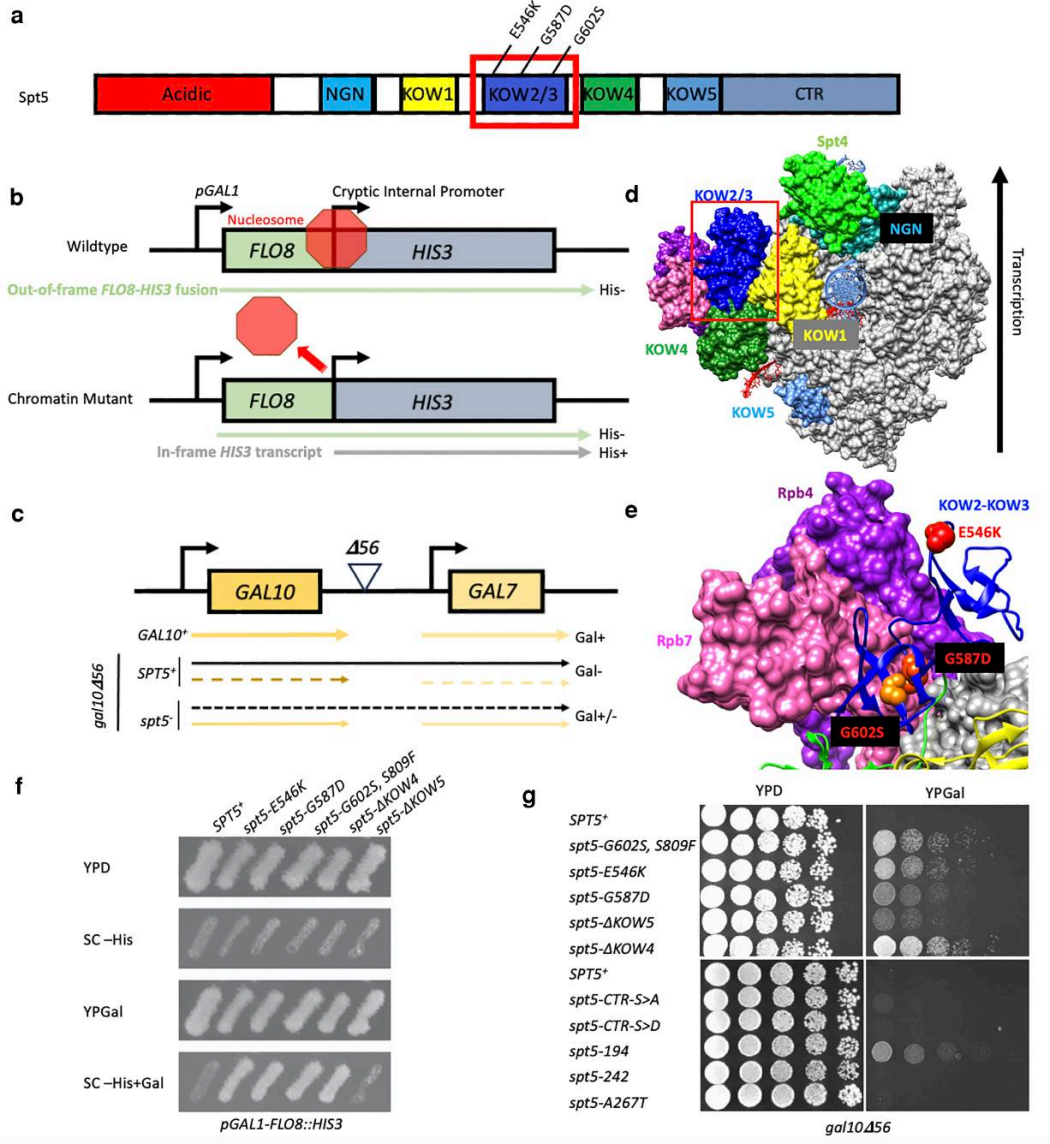
*Spt5* CI mutants lie adjacent to the Pol II stalk and suppress *gal10****Δ****56.* a) Spt5 domain architecture with cryptic initiation mutations indicated. b) Schematic of cryptic initiation reporter. Growth on –His media indicates initiation from internal promoter, which arises from altered chromatin structure. Reporter is under control of the *GAL1* promoter and is therefore inducible with addition of Galactose. c) Schematic of *gal10****Δ****56* reporter. Growth on galactose media indicates suppression of *gal10****Δ****56.* Suppression of galactose sensitivity arises from loss of inhibition of *GAL7* promoter due to reduced transcriptional interference from *GAL7* poly(A) readthrough. d) cryo-EM structure (PDB: 5OIK) of Pol II EC reconstructed from *B. taurus* Pol II and *H. sapiens* Spt4/5, with KOW2-3 region boxed. Image generated in ChimeraX (Bernecky et al. 2017). e) Zoomed in image of boxed portion of panel c, indicating amino acid changes of cryptic initiation mutants (PDB: 5OIK) (Bernecky et al. 2017). Image generated in ChimeraX. f) Haploid yeast *spt5***Δ** shuffle strain harboring the cryptic initiation reporter (GHY2741) was transformed with the indicated alleles of *spt5* on a *LEU2* centromeric plasmid and plated onto SC–Leu to select for successful transformation. Transformants were replica plated to 5-FOA to counter-select for loss of the wildtype *SPT5  URA3* plasmid. Single colonies were patched onto YPD, replica plated onto the indicated media and incubated at 30 °C. g) A haploid yeast *spt5***Δ** shuffle strain (GHY3246) with *gal10****Δ****56* harboring a wildtype *SPT5  URA3* plasmid was transformed with the indicated alleles of *SPT5* on a centromeric plasmid harboring *LEU2.* Successful transformants were selected for by plating onto SC—Leu, and subsequently replica plated to 5-FOA to counter-select for loss of the wildtype *SPT5  URA3* plasmid. Individual colonies were grown in YPD overnight and serial 5-fold dilutions were spotted onto YPD and YPGal plates and incubated at 30 °C.

We screened for *spt5* mutations that disrupt chromatin structure by utilizing the well-established galactose-inducible *pGAL1-FLO8::HIS3* cryptic initiation reporter, in which disruption of chromatin reveals an intragenic transcription start site that allows for growth on –His + Gal media (Cheung et al. 2008) (Fig. 1b). This screen yielded 3 mutations that alter residues within the KOW2-3 region of Spt5: G587D, G602S + S809F, and E546K (Fig. 1a and f). Spt5-G587 and -G602 pack closely at the juncture of Rpb7 and KOW3, while Spt5-E546 lies at the tip of KOW2 (Fig. 1d and e). Further characterization of these mutants and others identified from this screen will be described elsewhere. To determine if other KOW domains may also share this phenotype, we assessed two deletion constructs: *spt5-ΔKOW4 and spt5-ΔKOW5.* We found that while *spt5-ΔKOW4* did confer cryptic initiation, *spt5-ΔKOW5* did not (Fig. 1f).

Structural and biochemical studies of Pol II ECs show Spt5 KOW domains 2–4 lie adjacent to and contact Rpb4/7 (Fig. 1d) (Li et al. 2014; Bernecky et al. 2017; Ehara et al. 2017; Gong and Li 2023). Since 2 of the mutations we identified lie at the interface of Spt5 and Rpb7, we therefore hypothesized that the function of Spt5′s central KOW domains may be related to the known functions of Rpb4/7. Since loss of *RPB4* results in alteration of poly(A) site choice, we wondered if our KOW2-3 mutations would also alter poly(A) site choice (Runner et al. 2008; Moqtaderi et al. 2026). To test this, we utilized the *gal10**Δ**56* reporter gene (Kaplan et al. 2005) (Fig. 1c). This reporter truncates the *GAL10* poly(A) site, resulting in transcriptional readthrough into and interference of the *GAL7* promoter and yielding poor growth on galactose due to an inability to metabolize galactose-1-phosphate, a toxic intermediate of the galactose metabolic pathway. Mutations that perturb polyadenylation result in termination of *GAL10* at the truncated poly(A) site and restore growth on galactose media through relief of transcription initiation inhibition of the *GAL7* promoter. All 3 point mutations in KOW2-3 resulted in suppression of *gal10Δ56*, as did deletions of KOW4 and, to a lesser degree, KOW5 (Fig. 1g). To confirm that this functionality is specific to the KOW domains, we also tested previously described mutations in the N- and C-terminal regions of Spt5. With the exception of *spt5-194*, which has previously been shown to destabilize Spt5′s overall structure (Ding et al. 2010), these *spt5* mutations did not suppress *gal10**Δ**56*.

### Mutations in *rpb4/7* result in altered poly(A) site choice, cryptic initiation, and genetically interact with *spt5-KOW* mutants

**Fig. 2.**
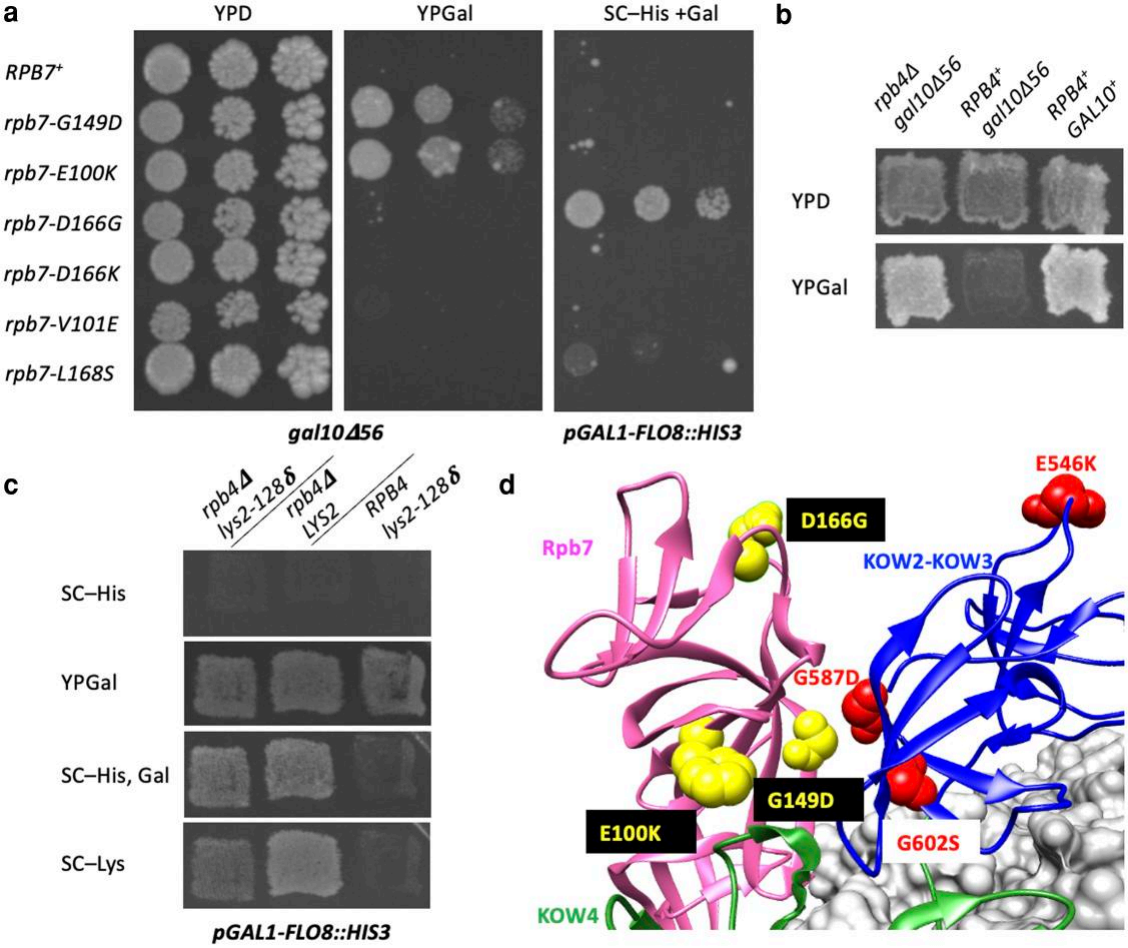
Mutations in *rpb4/7* result in cryptic initiation and suppression of *gal10Δ56*. a) Haploid yeast *rpb7* shuffle strains containing either *gal10**Δ**56* (GHY1555) or the cryptic initiation reporter (GHY3244) were transformed with the indicated alleles of *rpb7* on a *LEU2* centromere plasmid and plated onto SC–Leu to select for successful transformation. Transformants were replica plated to 5-FOA to counter-select for loss of the *RPB7  URA3* plasmid. Resulting colonies were grown in YPD overnight at 30 °C. Serial 5-fold dilutions were spotted onto the indicated media and incubated at 30 °C. b) Patches of haploid yeast strains harboring the indicated alleles of *RPB4* and *GAL10* (GHY4005, GHY3147, GHY1934) were grown on the indicated media at 30 °C. c) Patches of haploid yeast strains (GHY4009, GHY4008, and GHY1934) harboring the cryptic initiation reporter gene construct and the indicated alleles of *RPB4* and *LYS2,* grown on the indicated media at 30 °C. Growth of *lys2-128δ* strains on –Lys media indicates an Spt^−^ phenotype. d) Cryo-EM representation of *spt5* and *rpb7* alleles mapped to the Pol II EC (PDB: 5OIK) (Bernecky et al. 2017).

We next asked if *rpb4/7* mutations share phenotypes with *spt5-KOW2-3* mutations. As *RPB7* is an essential gene, we mutagenized plasmid-borne *RPB7* and screened for cryptic initiation and suppression of *gal10**Δ**56* via plasmid shuffle (Greger and Proudfoot 1998; Kaplan et al. 2005). Two mutations resulted in strong suppression of *gal10Δ56—rpb7-G149*D, and *rpb7-E100K* (Fig. 2a). Rpb7-G149 lies at the juncture of Rpb7 and KOW3 and sits near Spt5-G587, while Rpb7-E100 lies at the interface of Rpb7 and Spt5-KOW4 (Fig. 2d). We did not identify *rpb7* alleles that caused cryptic initiation in this screen. However, we also tested *rpb7* alleles generously provided by Craig Kaplan, which have been shown to exhibit transcriptional defects (*rpb7-D166G, rpb7-L168S, rpb7-V101E)* for suppression of *gal10Δ56* as well as cryptic initiation (Braberg et al. 2013). One of these alleles, *rpb7-D166G*, demonstrated strong cryptic initiation (Fig. 2a). Similar to Spt5-E546, Rpb7-D166 is a solvent-exposed residue that projects away from the polymerase (Fig. 2d). *Rpb7-D166G* has also been shown to result in transcription start site (TSS) defects as well as sensitivity to mycophenolic acid (MPA). Prior literature suggests that MPA sensitivity arises from defects in transcription initiation through altered TSS selection at *IMD2* (Malik et al. 2017). Additionally, Rpb7-D166 interacts directly with Tfb3, a component of TFIIH, providing a structural basis for its role in transcription initiation and MPA sensitivity (Braberg et al. 2013; Basnet et al. 2026). Furthermore, *rpb7-L168S* exhibited weak cryptic initiation, while *rpb7-V101E* did not suppress *gal10**Δ**56* or show cryptic initiation. This difference suggests that the cryptic initiation phenotypes seen in *rpb7-D166G* and *-L168S* are allele-specific, rather than resulting from general loss of function. Since Rpb7-D166G abolishes the negative charge of aspartate, we reasoned that a charge reversal may result in a stronger phenotype. We therefore engineered *rpb7-D166K* via PCR mutagenesis. However, contrary to our expectations, we found this allele did not give any observable phenotype as a single mutant.

To assess the evolutionary and functional significance of these Spt5 and Rpb7 residues, we generated sequence alignments across model organisms and asked if these residues were conserved (Supplementary Fig. 1). Spt5-G602 is very highly conserved as are the closely spaced Spt5-G587 and Rpb7-G149 residues. This suggests that the interface between Rpb7 and Spt5-KOW2-3 is evolutionarily relevant. Both Spt5-E546, Rpb7-D166, and Rpb7-L168 are also highly conserved across the species analyzed, highlighting the evolutionary importance of the surface-exposed structure formed by the C-terminus of Rpb7 and Spt5-KOW2.

Since *RPB4* is not essential, we deleted *RPB4* in strains carrying the cryptic initiation and *gal10**Δ**56* reporters and observed both suppression of *gal10**Δ**56* and cryptic initiation (Fig. 2b and c).

**Fig. 3.**
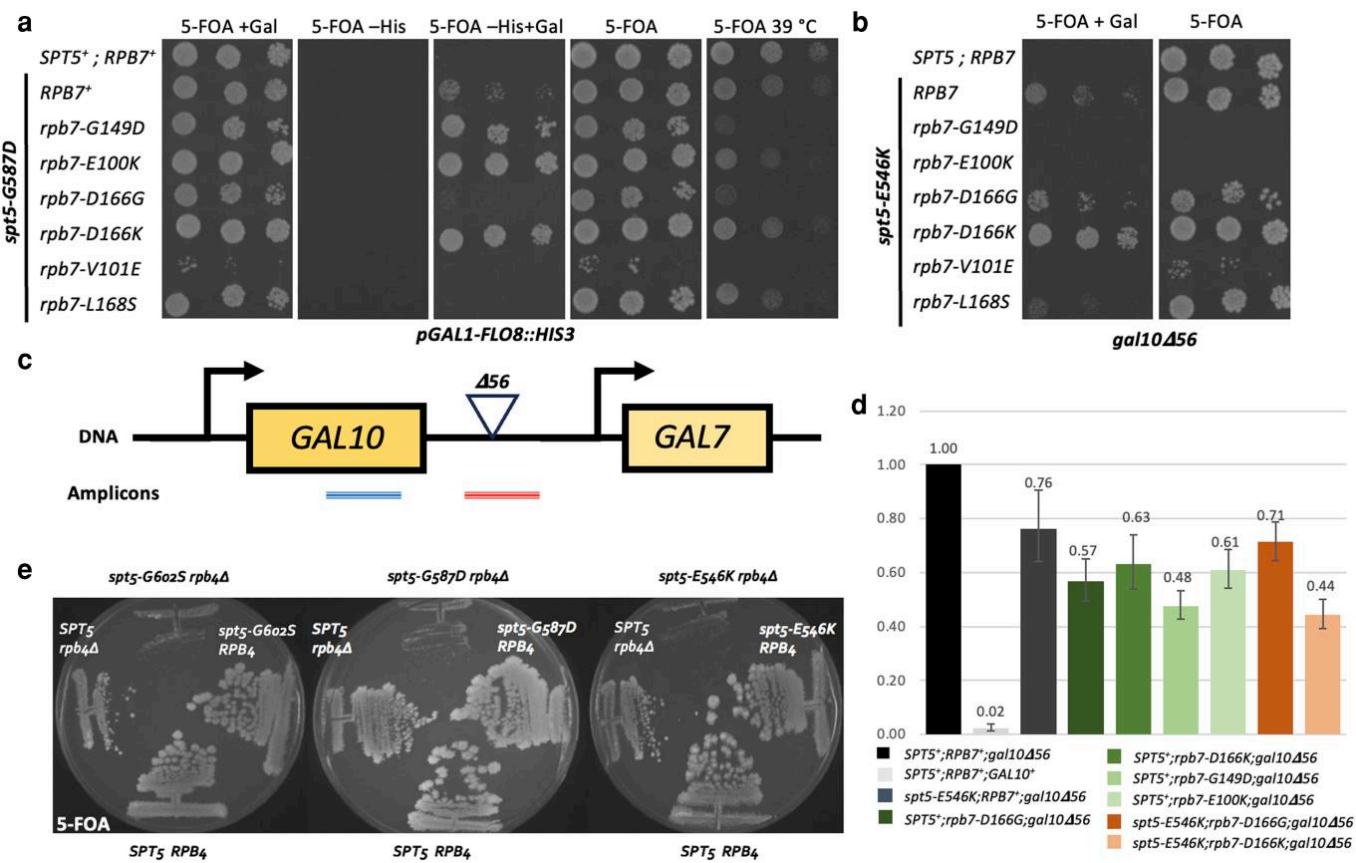
*SPT5* KOW2-3 and *RPB4/7* genetically and functionally interact. a) Serial 5-fold dilutions of *rpb7  spt5-G587D* double mutants. A haploid *rpb7***Δ**  *spt5-G587D* strain with the cryptic initiation reporter and an *RPB7  URA3* plasmid (GHY3348) was transformed with centromeric *rpb7  LEU2* plasmids carrying the indicated alleles of *RPB7* and plated onto SC –Leu media to select for uptake of the mutant plasmid. Transformants were grown in YPD overnight and serial 5-fold dilutions were spotted directly onto the indicated media containing 5-FOA to select for loss of the *RPB7  URA3* plasmid. A haploid *rpb7***Δ**  *SPT5+* strain harboring a wildtype *RPB7  LEU2* plasmid was used as a control (GHY3243). Spots were incubated at 30 °C unless indicated otherwise b) Serial 5-fold dilutions of *rpb7  spt5-E546K* double mutants. A haploid yeast *rpb7***Δ** shuffle strain with *spt5-E546K* and *gal10**Δ**56* (GHY3349) was transformed with centromeric *rpb7  LEU2* plasmids carrying the indicated alleles of *RPB7* and plated onto SC–Leu media to select for uptake of the mutant plasmid. Transformants were grown in YPD overnight. Serial 5-fold dilutions were spotted onto the indicated media and allowed to grow at 30 °C. A haploid *rpb7***Δ**  *SPT5  gal10**Δ**56* strain (GHY1555) harboring a wildtype *RPB7  LEU2* plasmid was used as a control. c) Schematic detailing location of PCR amplicons on the *gal10**Δ**56* gene construct. The upstream amplicon is within *GAL10* ORF, the downstream amplicon spans the *GAL10* poly(A) site. d) Bar chart indicating mean of *n* = 3 biological replicate **ΔΔ**Ct values ± SD of *GAL10* readthrough product normalized to total *GAL10* transcripts relative to *SPT5  RPB7  gal10**Δ**56*. Significance between *gal10**Δ**56; SPT5; RPB7* and all other backgrounds was calculated by 2-tailed Student's t-test; all *P*-values are < 0.002. Error bars indicate SD. e) Haploid *spt5***Δ** shuffle strains harboring either wildtype *RPB4* (GHY2741) or *rpb4***Δ** (GHY3248) and *SPT5  URA3 CEN* plasmids were transformed with the indicated alleles of *SPT5* on a *LEU2 CEN* plasmid and plated onto SC–Leu media to select for transformants. Transformants were struck out on –Leu media and replica plated to 5-fluoroorotic acid (5-FOA) for counterselection against the *SPT5  URA3* plasmid. Note: the *spt5-G602S-*containing plasmid also harbors *spt5-S809F.*

To further assess the functional cooperation of Spt5 KOW2-3 with the Pol II stalk, we generated double mutants of *spt5-E546K* and *spt5-G587D* with our previously identified *rpb7* mutants. Since *spt5-G602S* is also paired with *spt5-S809F*, we excluded this mutant from analysis as it would confound mechanistic interpretations of our results. We first tested our *rpb7* alleles in combination with *spt5-G587D* in the cryptic initiation background. *Spt5-G587D*, which displays a strong cryptic initiation phenotype when plasmid-borne (Fig. 1f), showed a moderately attenuated phenotype when integrated. When combined with *spt5-G587D*, we observed a strong synthetic cryptic initiation phenotype in *rpb7-G149D*, *-E100K*, and *-D166K* (Fig. 3a). Interestingly, the cryptic initiation phenotype observed in the *rpb7-D166G* single mutant (Fig. 2a) was suppressed in the presence of *spt5-G587D*. Additionally, we observed strong synthetic growth defects at 39 ° C when *spt5-G587D* was combined with *rpb7-G149D, -D166G and -V101E*.

We next tested *rpb7* alleles in combination with s*pt5-E546K* in the *gal10**Δ**56* background (Fig. 3b). There was little if any additional effect when *spt5-E546K* was paired with *rpb7-D166G*; however, the suppression of *gal10**Δ**56* was clearly enhanced when paired with *rpb7-D166K*. In contrast, suppression of *gal10**Δ**56* was reduced when *spt5-E546K* was combined with *rpb7-L168S*, and *spt5-E546K* exhibited no detectable growth when combined with *rpb7-G149D* or -*E100K,* consistent with loss of viability. The *spt5-E546K, rpb7-V101E* double mutant displayed severely impaired growth, with only a small number of slow growing colonies observed across replicates. When we combined *spt5-G602S + S809F*, *spt5-E546K*, or *spt5-G587D* with a deletion of *RPB4*, we observed that all 3 double mutants were inviable after 5-FOA passage (Fig. 3e). Prior to passage over 5-FOA, we observed that the *rpb4**Δ**  spt5-E546K* and *spt5-G587D* double mutants grew more poorly than *rpb4**Δ*** alone, indicating a dominant growth defect (Supplementary Fig. 2). A detailed summary of the phenotypes of all *spt5, rpb7* and *rpb4* alleles analyzed in this study is available in Supplementary Table 4.

### Molecular readthrough at *GAL10* corroborates altered poly(A) site choice

To further investigate the genetic suppression of *gal10**Δ**56* observed in *spt5* and *rpb7* mutants, we utilized RT-qPCR to quantify readthrough of *GAL10* mRNA in *gal10**Δ**56* strains. We designed 2 sets of primers. The first falls within the *GAL10* ORF and will detect both readthrough and normally terminated *GAL10* transcripts. The second primer pair spans the *gal10**Δ**56* mutation and will only detect readthrough transcripts (Fig. 3c). To calculate the ratio of *GAL10* transcripts that fail to terminate properly, we calculated the ratio of the readthrough product to total *GAL10* mRNA normalized to the readthrough:total GAL10 ratio measured in a *SPT5  RPB7  gal10**Δ**56* strain. A ratio < 1 indicates reduced readthrough. We found that *spt5-E546K* resulted in 0.76-fold readthrough relative to wild type*, rpb7-D166G* resulted in 0.57-fold readthrough, and -*D166K* resulted in 0.63-fold readthrough relative to wild type (Fig. 3d). We found that the *rpb7*-*G149D* and -*E100K* alleles resulted in a significant 0.48- and 0.61-fold reduction in readthrough, reflective of their strong suppression of *gal10**Δ**56.* We further found that when combined with *spt5-E546K, rpb7-D166G* resulted in 0.71-fold readthrough relative to wild type, which is consistent with the lack of additive effects seen in the genetic assay. *Spt5*-*E546K* + *Rpb7-D166K* showed significantly reduced readthrough that was lower than either single mutant alone at 0.44-fold, which also reflects the enhanced suppression in the genetic assay. Interestingly, while *rpb7-D166G* resulted in similar reduction in readthrough to other mutants, it did not suppress *gal10**Δ**56* on its own. Aside from this exception, the molecular results observed in our RT-qPCR assay are in close alignment with the genetic suppression observed with *gal10**Δ**56.*

### Spt5 KOW pulldown proteomics enrich factors from CPF/CF and NNS termination complexes as well as chromatin related factors

Proteomic and biochemical studies have identified a number of factors that interact with Spt5 (Krogan et al. 2002; Lindstrom et al. 2003). However, these binding events have not been assigned to individual Spt5 domains. Since Spt5′s domains are widely distributed across the surface of Pol II, it is reasonable to suspect that the different Spt5-interacting factors may bind exclusively to specific domains of Spt5 to support location-specific roles. Supporting this hypothesis, Spt5′s KOW domains are composed of Tudor folds which, in other proteins, are known to support protein–protein and protein–nucleic acid interactions (Lasko 2010; Meyer et al. 2015). To pinpoint the function of the conjoined Spt5 KOW-stalk region of the elongation complex, it is therefore necessary to identify factors that bind specifically to the KOW domains that participate in this structure.

To accomplish this, we performed affinity chromatography followed by MudPIT mass spectrometry to identify proteins captured by either purified KOW2-3 (K2K3) region, Linker2-KOW4 (L2K4) region, or BSA. To this end, we purified recombinant His-tagged K2K3 and L2K4 and BSA and immobilized them on Affi-Gel. The column was then challenged with a clarified yeast lysate, washed, and eluted via salt gradient. Silver-stained gels of the eluates revealed that the KOW2-3 and L2K4 bound noticeably different patterns of proteins (Supplementary Fig. 3). The 3 highest salt fractions from K2K3, L2K4 and BSA were subjected to mass spectrometry.

**Fig. 4.**
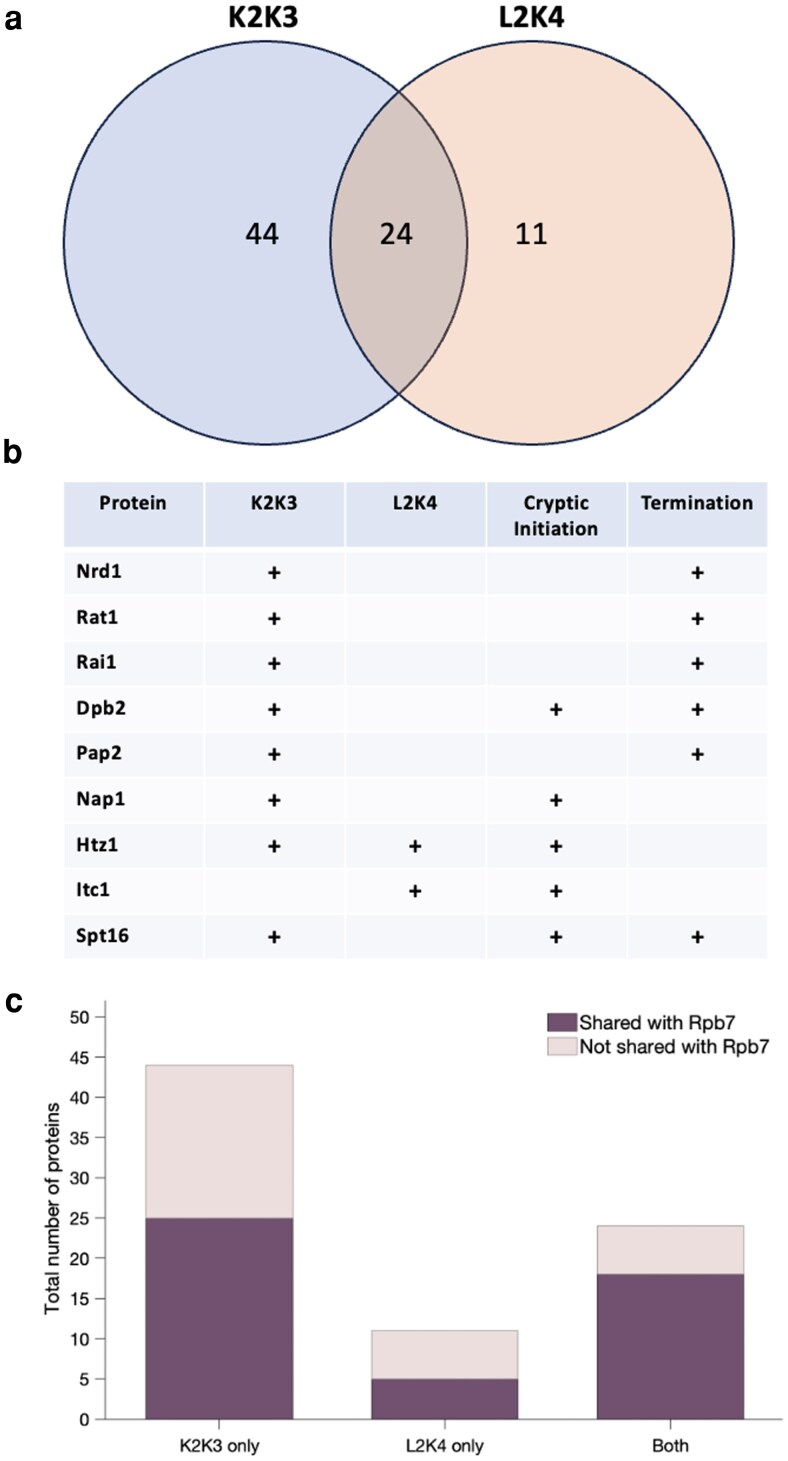
KOW domain interactomes overlap with Rpb7-associated factors and are enriched for factors associated with chromatin regulation and 3′-end processing. a) Venn Diagram highlighting overlap between factors enriched in K2K3 and L2K4 MudPIT mass spectrometry datasets. b) Curated table of select factors enriched in K2K3 and L2K4 datasets that overlap with known Rpb7 interactors and have known functions in repressing cryptic initiation and regulating 3′-end processing (Steinmetz et al. 2001; Scorilas 2002; Kaplan et al. 2003; Arigo et al. 2006; Luo et al. 2006; Cheung et al. 2008; Xiang et al. 2009; Cloutier et al. 2012; Schmidt et al. 2012; Yen et al. 2012; Xue et al. 2013; Jeronimo et al. 2015; Campbell et al. 2019; Aydin et al. 2024; Bandyopadhyay et al. 2025; Moqtaderi et al. 2026). c) Stacked bar chart indicating degree of overlap between factors enriched in K2K3 and L2K4 datasets and previously identified Rpb7-associated factors (Mitsuzawa et al. 2003; Mosley et al. 2013).

We identified many proteins that were differentially enriched by KOW2-3 and L2K4, as well as proteins that bound both domains. We excluded a number of proteins from analysis: ribosomal proteins, which are frequently identified nonspecifically in mass spectrometry, cytoplasmic proteins, as well as importins and mitochondrial membrane proteins. Spt5 is known to interact with RNA Polymerase I, and we did identify a number of RNA Polymerase I subunits; however, we excluded ribosome biogenesis factors as this study focuses on Pol II transcription. Finally, any proteins that were enriched less than two-fold relative to the BSA control column were excluded. These exclusions left 79 proteins that were enriched in either K2K3, L2K4, or both (Fig. 4a, Table 1). This list includes proteins involved in a wide array of nuclear functions, including transcription, DNA replication, and splicing. Consistent with the cryptic initiation phenotype observed in our *rpb4/7* and *spt5* KOW2-3 mutants, we also identified proteins involved in chromatin structure and 3′-end formation, including Nap1, Nbp2, Histones H3, H2A.Z, H2B, and Spt16. Interestingly, we also identified factors in both NNS and CPF/CF termination pathways.

**Table 1.** Identification of proteins that interact with Spt5′s central KOW domains.

Protein type and name	Notes	Domain of Spt5	Protein type and name	Notes	Domain of Spt5
**Pol subunits**			**Transcription**		
**Rpc10**	RNAPI, II, III subunit	K2K3, L2K4	Ncb2	Subunit of NC2 complex	K2K3
Rpc19	RNAPI, RNAPIII subunit	K2K3	**Elp3**	Elongator subunit	K2K3, L2K4
**Rpa49**	RNAPI subunit	K2K3	**Elp2**	Elongator subunit	K2K3, L2K4
**Rpa135**	RNAPI subunit	K2K3	**Iki3**	Elongator subunit	K2K3, L2K4
**Rpa190**	RNAPI subunit	K2K3	Elp4	Elongator subunit	K2K3
Rpa34	RNAPI subunit	K2K3	**Dig1**	Inhibitor of Ste12	K2K3
**Ret1**	RNAPIII subunit	K2K3	**Spt5** ^a,b^	Transcription elongation	K2K3, L2K4
**Rpb8**	RNAPI, II, III subunit	K2K3	Brf2	TFIIIB B-related factor	K2K3, L2K4
Rpc344	RNAPIII subunit	K2K3			
**Termination**			**Replication**		
**Nrd1** ^a^	Subunit of Nrd1 complex	K2K3	**Smc1**	Cohesin subunit	K2K3
Nab3	Subunit of Nrd1 complex	K2K3	Rfc3	Replication factor C subunit	K2K3, L2K4
**Rat1** ^a^	ssRNA exonuclease	K2K3	**Rfc1**	Replication factor C subunit	K2K3, L2K4
**Rai1** ^a^	Rat1 binding	K2K3	**Rfc2**	Replication factor C subunit	K2K3, L2K4
**Dbp2** ^a,b^	RNA Helicase	K2K3	**Rfc5**	Replication factor C subunit	K2K3, L2K4
**RNA Processing**			Rfc4	Replication factor C subunit	K2K3, L2K4
Csl4	Exosome component	K2K3	**Other**		
**Rok1**	RNA helicase	K2K3	**Ppn1**	Endopolyphosphatase	K2K3, L2K4
**Pap2** ^a^	Component of TRAMP	K2K3	**Ckb1**	Casein kinase II subunit	K2K3
Air1	Component of TRAMP	K2K3	**Cka2**	Casein kinase II subunit	K2K3
**Dbp3**	RNA helicase	K2K3, L2K4	**Cka1**	Casein kinase II subunit	K2K3, L2K4
Xrn1	5′-3′ exoribonuclease	K2K3, L2K4	Hrr25	Casein kinase I homolog	K2K3
Gbp2	Poly(A+) binding protein	K2K3, L2K4	**Ckb2**	Casein kinase II subunit	K2K3, L2K4
**Mtr4**	Component of TRAMP	K2K3	**Sip5**	Snf1-interacting protein	L2K4
**Sro9**	RNA-binding protein	K2K3	Yhp1	Transcriptional repressor	L2K4
**Ski2**	SKI complex component	K2K3, L2K4	**Cdc13**	ssDNA-binding protein	L2K4
**Ski3**	SKI complex component	K2K3, L2K4	**Pho81**	CDK inhibitor	L2K4
Ski8	SKI complex component	K2K3, L2K4	TY1B-LR3, JR1, PR3	TY1-LR3 Gag-Pol polyprotein	L2K4
**Chromatin**			TY1B-ER2, LR1, MR2	TY1-LR2 Gag-Pol polyprotein	L2K4
**Nap1** ^b^	Histone chaperone	K2K3	TY1B-PR3	TY1-PR3 Gag-Pol polyprotein	K2K3
Nbp2	Nap1 binding protein	K2K3	TY1B-NL1	TY1-NL1 Gag-Pol polyprotein	K2K3
Hht1	Histone H3	L2K4	**Sbp1**	Found in P-bodies	K2K3
**Htz1** ^b^	Histone H2A.Z	K2K3, L2K4	**Pby1**	Associated with P-bodies	K2K3
Htb2	Histone H2B.1,	L2K4	**Ptc1**	Dephosphorylates Hog1	K2K3
**Fpr3**	nucleosome assembly	K2K3, L2K4	YML108W	Unknown Function	K2K3
**Fpr4**	Nucleosome assembly	K2K3, L2K4	Pci8	Cop9 component	K2K3
**Itc1** ^b^	Isw2-Itc1 subunit	L2K4	**Tyw1**	Wybutosine synthesis	K2K3
Euc1	DNA binding protein	K2K3	**Sda1**	Binds Nap1	K2K3
**Spt16** ^a,b^	FACT complex subunit	K2K3	**Kip3**	Motor protein	K2K3
**Splicing**			**Msh1**	Mitochondrial DNA repair	K2K3
**Prp43**	RNA helicase	L2K4	**YCR051W**	Ankyrin-repeat containing	K2K3
Lsm5	U6 Subunit	L2K4	**Mak5**	DEAD-box RNA helicase	K2K3, L2K4
Lsm3	U4/U6-U5 subunit	K2K3	Hcr1	EIF3 component	K2K3

List of proteins identified via MudPIT mass spectrometry that are enriched at least 2-fold relative to BSA in pull-downs against the indicated KOW domain, excluding cytoplasmic proteins, ribosomal biogenesis factors, importins and mitochondrial proteins from the 1 M salt fraction. Proteins indicated in bold were previously identified as Rpb7-interacting (Mitsuzawa et al. 2003; Mosley et al. 2013). Underlined proteins were previously identified as Spt5-interacting (Lindstrom et al. 2003).

^a^Indicates factors that have previously been associated with regulating termination site choice.

^b^Indicates factors that have previously been associated with repression of cryptic transcription (Steinmetz et al. 2001; Scorilas 2002; Kaplan et al. 2003; Arigo et al. 2006; Luo et al. 2006; Cheung et al. 2008; Xiang et al. 2009; Cloutier et al. 2012; Schmidt et al. 2012; Yen et al. 2012; Xue et al. 2013; Jeronimo et al. 2015; Campbell et al. 2019; Aydin et al. 2024; Bandyopadhyay et al. 2025; Moqtaderi et al. 2026).

Prior proteomics studies and two-hybrid screens have identified factors that interact with Rpb7 (Mitsuzawa et al. 2003; Mosley et al. 2013). A substantial proportion of proteins enriched in our dataset overlap with those which were previously identified as Rpb7-interacting (Fig. 4c, Table 1). Notably, several factors enriched in our dataset and shared with previously reported Rpb7-associated factors are implicated in both transcription termination regulation as well as repression of cryptic transcription, strengthening the notion that this region participates in a common function (Fig. 4b).

Both Nrd1 and Nab3 (2 out of 3 components of the NNS noncoding termination complex) were enriched by KOW2-3 in our study, and Seb1 (the *S. pombe*  Nrd1 ortholog) was identified in prior studies as interacting directly with Rpb7-D167 (which aligns with Rpb7-D166 in *S. cerevisiae)*, indicating that the region comprised of Spt5 KOW2-3 and Rpb4/7 may be responsible for regulating NNS-dependent termination (Mitsuzawa et al. 2003; Mosley et al. 2013). Given that our prior analyses focused on poly(A)-dependent termination, this data motivated us to examine whether perturbation of this interface impacts NNS-dependent processes as well, which would indicate that this structure is a global regulator of termination rather than having a poly(A) specific function.

### Allele-specific regulation of NNS termination at *SNR13* by Spt5 and Rpb7

**Fig. 5.**
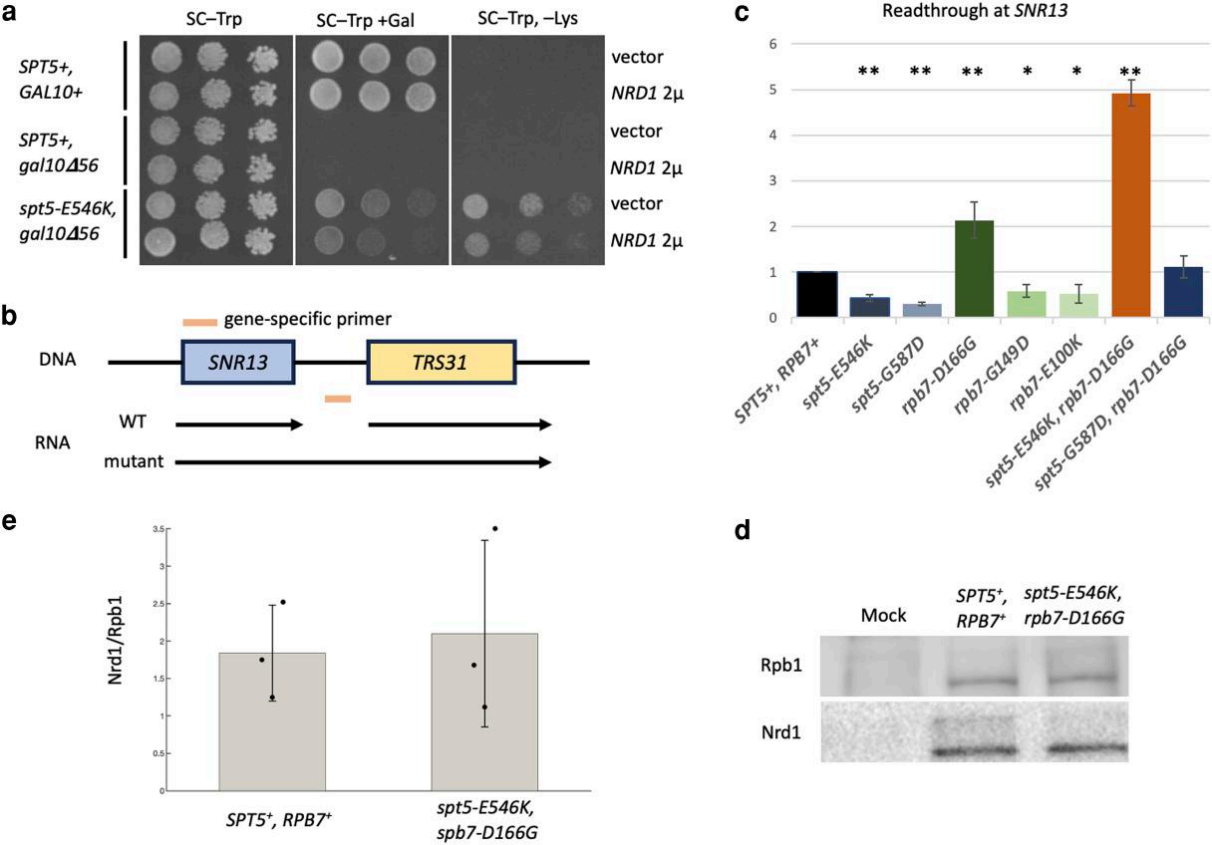
*Spt5/rpb7* mutants alter noncoding RNA termination and interact genetically with *NRD1* without disrupting binding to Pol II. a) Serial 5-fold dilutions of haploid yeast strains (GHY1934, GHY3147, GHY3335) with the indicated alleles of *GAL10* and *SPT5*, with and without *NRD1* supplied on a high-copy vector (2μ) were spotted onto the indicated media and incubated at 30 °C. b) Schematic showing locations of primers at the *SNR13, TRS31* locus. c) Bar chart showing mean of *n* = 3 biological replicate **ΔΔ**Ct values ± SD of readthrough product at *SNR13* normalized to *ACT1* compared to wildtype. Two-tailed Student's t-test was performed comparing WT to all other conditions to assess significance: * indicates *P* < 0.05, ** indicates *P* < 0.01. Error bars indicate SD. d) Western blot with equal inputs of Rpb3-TAP pull-down TEV-treated eluates in mock (untagged), WT and *spt5-E546K rpb7-D166G* genetic backgrounds, with primary antibody indicated on the left. Blots were stripped in between probing for Nrd1 and Rpb1. Representative of *n* = 3 biological replicates. Strains used were FY603, GHY3244, and GHY3368. GHY3368 was transformed with CKB310 and passed over 5-FOA prior to analysis. e) Quantification of Nrd1 signal normalized to Rpb1 from 3 biological replicates of Rpb3-TAP pull-downs. Individual data points are shown with mean ± SD.

Given the enrichment of Nrd1 in the KOW2-3 pulldown, we considered the possibility that the Spt5 KOW2-3/stalk region plays a role in NNS termination. To examine this possibility, we first asked if *NRD1* overexpression could modulate *spt5* mutant phenotypes. We transformed yeast harboring the *gal10**Δ**56* reporter and *spt5-E546K* with a *NRD1* overexpression vector and assayed for changes in phenotypes observed in the *spt5* single mutant. We observed modest suppression of both the *gal10**Δ**56* and the Spt^−^ phenotype in *spt5-E546K* (Fig. 5a). This suggests that Nrd1 dosage can influence *spt5* mutant phenotypes.

*Nrd1* mutations attenuate transcription termination at the snoRNA gene *SNR13*, resulting in accumulation of transcripts that extend beyond the normal termination site (Steinmetz et al. 2001; Lee et al. 2020). Using an RT-qPCR assay that was previously used to demonstrate *SNR13* readthrough due to NNS defects (Lee et al. 2020), we asked if mutations affecting the Spt5 KOW2-3/stalk cause a similar read-through defect. To detect transcripts that extend past the normal *SNR13* termination site, this assay amplifies cDNA with a forward primer positioned within *SNR13* and a reverse primer in the downstream intergenic region between *SNR13* and *TRS31* (Fig. 5b) (Lee et al. 2020). Normalization to ACT1 expression controlled for global changes in gene expression. However, this primer pair does not fully distinguish between increased readthrough or changes to stability of the transcript. Consistent with our expectations, we found that *rpb7-D166G* resulted in two-fold increase in signal relative to wild type (Fig. 5c). Furthermore, we found that the *rpb7-D166G, spt5-E546K* double mutant showed an approximately 5-fold increase relative to wild type. We also found that *spt5-E546K, spt5-G587D, rpb7-E100K* and *rpb7-G149D* resulted in decreased signal, indicating enhanced 3′-end formation, consistent with our observations in *gal10**Δ**56.* Interestingly, the *spt5-G587D, rpb7-D166G* double mutant resulted in suppression of readthrough to wild type levels, which is consistent with our observation that this double mutant resulted in suppression of cryptic initiation. To test if this double mutant disrupts recruitment of Nrd1 to the Pol II EC, we performed a pull-down experiment targeting TAP-tagged Rpb3 in wild type and mutant contexts probing for Nrd1 via Western blot. We found that there was no difference in the amount of Nrd1 associated with Pol II in the double mutant context compared to wild type, indicating that bulk Nrd1-Pol II association is retained (Fig. 5d and e).

## Discussion

As Pol II traverses the length of a gene, the stalk region and its interacting factors are likely remodeled to better serve the elongation complex in response to co-transcriptional challenges, such as chromatin, mRNP packaging, and 3′-end processing (Mosley et al. 2013). Part of this remodeling procedure may involve a shift in conformation of Spt5′s central KOW domains or transient dissociation of Rpb4/7. Prior studies have shown that Pol II ECs lacking Rpb4/7 are enriched for specific elongation factors, and Pol II ECs purified via affinity-tagged Rpb7 are depleted for serine-2 CTD phosphorylation, suggesting that Rpb4/7 may dissociate in response to specific signals during elongation (Mosley et al. 2013). In fact, recent studies of native Pol II purified from *D. melanogaster* embryos have elucidated the structure of a stalk-less elongation complex actively engaged with and transcribing DNA, supporting the idea that 10-subunit Pol II has *in vivo* functions (Venette-Smith et al. 2025). Our genetic analysis of *spt5* and *rpb7* mutants supports a model in which the spatial arrangement of Rpb7 and Spt5′s central KOW domains relative to each other is functionally relevant. Importantly, the consequences of perturbing this surface are allele-specific: while Rpb7-G149 and Spt5-G587 are adjacent to each other according to many cryo-EM models, *rpb7-G149D* did not share the same phenotype as *spt5-G587D.* If this were the only relevant conformation, one would expect that mutating either *RPB7* or *SPT5* would result in the same phenotype. However, we observe that *rpb7-G149D* only resulted in a *gal10**Δ**56* phenotype, while *spt5-G587D* confers defects both in *gal10**Δ**56* and cryptic initiation. This suggests that merely disrupting the interface between Spt5 and Rpb7 is not sufficient to induce cryptic initiation, and that Spt5-KOW2-3 may have an independent function when this interface is disrupted. Despite this separation of function, our observation that *spt5-E546K* is inviable in the presence of *rpb7-E100K* or *rpb7-G149D* argues that the Spt5-stalk surface functions cooperatively, and disruption of this surface compromises an essential cellular function. Whether the observed phenotypes are a result of induced dissociation of Rpb4/7 or conformational shifts of KOW2-4 will require future work. One possibility is that Spt5 contributes to retention or stabilization of the Pol II stalk during transcription.

Our finding that the *rpb7-D166G, spt5-E546K* double mutant does not impair recruitment of Nrd1 to Pol II is consistent with the existence of redundant recruitment mechanisms, including interactions with the Spt5 CTR and recognition of serine-5 phosphorylation of the Pol II CTD via the Nrd1 CID (Leporé and Lafontaine 2011; Heo et al. 2013). Although these mutations do not prevent NNS recruitment to Pol II, they may influence the productive engagement of NNS factors with the elongation complex or with nascent RNA. Previous work in *Schizosaccharomyces pombe* has shown that Rpb7-D167, which aligns with *S. cerevisiae*  Rpb7-D166, is required for maintaining contact with Seb1, the *S. pombe* ortholog of Nrd1, and this interaction is conserved in *Saccharomyces cerevisiae* (Mitsuzawa et al. 2003). This suggests that the phenotypes we observe may result from altered positioning of NNS factors relative to the KOW-stalk surface.

Mutations in *SPT6* and other elongation factors have been shown to enhance upstream poly(A) site utilization at *GAL10* and other loci (Cui and Denis 2003; Kaplan et al. 2005; Geisberg et al. 2024; Moqtaderi et al. 2026). One model to explain this phenotype is that defects in transcription elongation result in a slower, more pause-prone polymerase, allowing for an increase in processing time at a weakened poly(A) site. Indeed, polymerase speed has been correlated with differences in poly(A) profiles across many genes, with a slower polymerase favoring upstream poly(A) sites, and a fast polymerase favoring downstream poly(A) sites (Geisberg et al. 2020, 2022). Further, defects in chromatin-related proteins such as Spt6 and FACT have been shown to shift poly(A) site choice upstream through effects on elongation rate arising from chromatin perturbations (Geisberg et al. 2024). However, these observations cannot completely explain the alterations in poly(A) site choice at *GAL10.* Kaplan et al. (2005) reported that in an *spt6* mutant the upstream poly(A) site is equally favored over 2 downstream alternative poly(A) sites. For this phenotype to be strictly explained by a slower elongating Pol II at *GAL10*, one would expect a gradient of decreasing poly(A) site usage following the initial Poly(A) site. This suggests that an alternative model may be true: suppression of *gal10**Δ**56* by perturbing elongation factors is caused by an alteration in either conformation or composition of the elongation complex. Supporting this, recent cryo-EM experiments have elucidated the structure of Pol II bound with the exonuclease Rat1, its binding partner Rai1, and Spt5 (Zeng et al. 2024). Interestingly, Pol II complexes that resolved Rat1 and Rai1 only showed density for Spt5-KOW5. In these structures, Rat1 binds Rpb7 at the same general location as Spt5 KOW2-3 and appears to contact Rpb7-G149, suggesting that Rat1/Rai1 trigger eviction of Spt5 KOW2-3 without disrupting general Spt5 association to Pol II. Single-molecule studies have also shown that Spt4/5 reversibly dissociates from the EC during a single transcription cycle (Rosen et al. 2020). These observations support the model that Spt5′s central KOW domains may indeed shift in conformation in response to the needs of the transcription complex.

Taken together, these results suggest that the *spt5* and *rpb7* mutations may alter the functional interaction between Spt5 KOW2-3 and the stalk. Interestingly, *rpb7-G149D* was recently independently isolated in an unbiased screen for mutations that derepress TCR in a TCR-defective background (Gong and Li 2023). In this context, derepression of TCR refers to increased or unmasked TCR activity under conditions in which it is normally inhibited. In this study, the authors utilized photoreactive crosslinking to probe interactions between Rpb7 and Spt5 in wildtype and Rpb7-G149D backgrounds. In the mutant context, they observed reduced crosslinking between Rpb7-E148, Rpb7-I151, and Spt5, consistent with altered positioning at this surface. They also observed disruption of crosslinking between Rpb7-E100 and Spt5 in Rpb7-G149D, potentially explaining why *rpb7-G149D* and *rpb7-E100K* mirror each other's phenotypes. Previous studies have shown that *spt4***Δ** suppresses TCR defects in *rad26***Δ** strains, indicating that loss of Spt4 shifts the Pol II EC into a state that is intrinsically competent for TCR (Jansen et al. 2000). Further, evidence suggests that Spt4-mediated TCR derepression may involve destabilization of Spt5 from Pol II (Ding et al. 2010). Together, these findings support a model in which perturbation of the Spt5 KOW2-3/Rpb4/7 interface shifts the Pol II EC toward alternate functional states that favor 3′-end formation and TCR.

This work has identified a potentially novel function for the Pol II stalk in chromatin regulation through characterization of alleles that result in cryptic initiation. The identification of novel alleles in *spt5* that also result in cryptic initiation near the Pol II stalk, as well as enrichment of Spt16, Nap1 and histone proteins in KOW pulldowns, supports a model in which this surface is involved in regulating chromatin structure during transcription. One prominent feature of histone interacting proteins is the presence of acidic regions that promote interactions with the basic histone proteins. The surface composed of Rpb4/7 and KOW2-3 is predominantly acidic in nature according to a charge distribution map (Supplementary Fig. 4), and the surface-exposed residues altered in our cryptic initiation assay are both acidic (Rpb7-D166, Spt5-E546), suggesting this surface has the potential to engage histones. Interestingly, a recent structural model of isolated native Pol II ECs shows Rpb7 directly contacting an upstream nucleosome (Kujirai et al. 2025). In this structure, Rpb7-D166 lies nearby, but not in direct contact with the incoming nucleosome. Spt5 and other elongation factors were not resolved in this structure, and the authors interpreted this to be evidence of elongation factor-independent nucleosome reassembly via template looping. The authors note, however, that elongation factors may have dissociated during sample preparation. An alternative model for the cryptic initiation phenotype we observed is that KOW2-3 and Rpb4/7 do not directly facilitate nucleosome passage themselves but form a platform for the assembly of other chromatin modifying factors, thereby exerting an indirect effect on chromatin structure. Supporting this model, our mass spectrometry data identified the histone chaperones Nap1 and Spt16 as enriched in the KOW2-3 pulldown. Further, recent structural evidence shows Spt6′s cognate binding partner Iws1/Spn1 binding to Spt5′s NGN and KOW2 domains simultaneously with Spt6′s N-terminal helices (Ehara et al. 2022). Additionally, Spt6 has been shown through structural studies to make extensive contacts with Rpb4/7 (Ehara et al. 2017; Vos et al. 2018). Notably, these structures show contacts between Spt6 and Rpb7-D166, as well as Spn1 and Spt5-E546, suggesting a potential mechanism for chromatin disruption in *rpb7-D166G* and *spt5-E546K.* Our results, together with emerging evidence suggest that chromatin structure and transcription termination are mechanistically coupled (Moqtaderi et al. 2026). Our observation that mutations within the KOW-stalk surface produce defects in both processes supports this model. Future experiments should distinguish direct from indirect roles of Spt5 and Rpb7 in nucleosome traversal and reassembly, for example using single-molecule optical tweezers assays (Chen et al. 2019; Burgos-Bravo et al. 2025).

Taken together, these results support a model in which the surface formed by Spt5 KOW2-3 and the Pol II stalk regulates chromatin integrity and termination in a context-dependent manner. Future studies should consider that this structure acts to provoke interactions with tertiary factors to execute these functions. This model does not preclude, however, direct effects on chromatin or RNA, either via direct interactions with nucleosomes through its acidic surface, or direct contacts with RNA to regulate termination outcome, as the stalk and Spt5 have both been shown to bind RNA (Meka et al. 2005; Ujvári and Luse 2006; Blythe et al. 2016; Zuber et al. 2018). Future work should aim to deconvolute direct vs indirect effects of this surface on co-transcriptional processes and define the contexts in which these mechanisms are regulated.

### Limitations of this study

The affinity chromatography/MudPIT experiments were performed using isolated Spt5 domains and a whole-cell yeast lysate and likely capture both direct as well as indirect associations. While factors involved in transcription and RNA processing were enriched, Pol II-specific subunits were not prominently recovered, and several RNA Polymerase I (Pol I) specific subunits were detected, consistent with the known association of Spt5 with Pol I transcription complexes, and may reflect the relative abundances or stabilities of Pol I and Pol II. The mass spectrometry results therefore do not differentiate between Pol I- and Pol II-associated interactions and are representative of Spt5′s association with co-transcriptional networks.

## Supplementary Material

jkag123_Supplementary_Data

## Data Availability

All strains and plasmids are available upon request. The authors affirm that all data necessary for confirming the conclusions of the article are present within the article, figures, and tables. A detailed list of yeast strains used throughout this manuscript is available in Supplementary Table 1. A detailed list of plasmids used is available in Supplementary Table 2. A list of all oligonucleotides used in this study is available in Supplementary Table 3. A complete list of proteins identified in MudPIT mass spectrometry experiments are available as File S1 (K2K3), File S2 (L2K4), File S3 (BSA). The raw data have been deposited to the MassIVE repository with the dataset identifier MSV000101465 Supplemental material available at G3 online.
