# Supplementary figures and images for: Spt5′s central KOW domains and the Pol II stalk collaborate to regulate chromatin and 3′-end processing in Saccharomyces cerevisiae
Source: G3 (Bethesda). 2026 May 11;16(7):jkag123. doi: 10.1093/g3journal/jkag123 (PMC13334181; doi:10.1093/g3journal/jkag123)

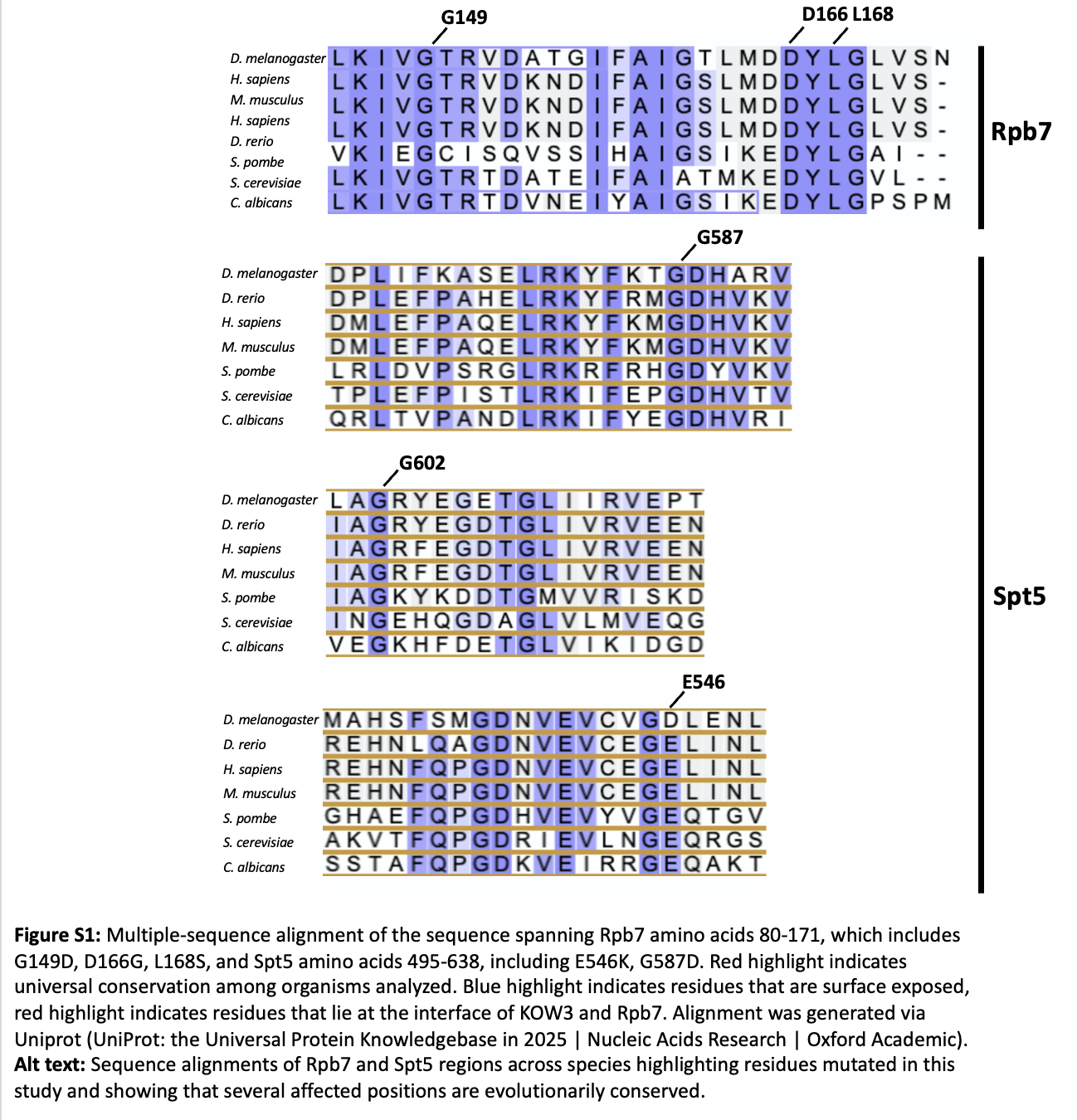

Supplement: jkag123_Supplementary_Data [file jkag123_supplementary_data.zip › Figure_S1_G3-2026-406685.tif]

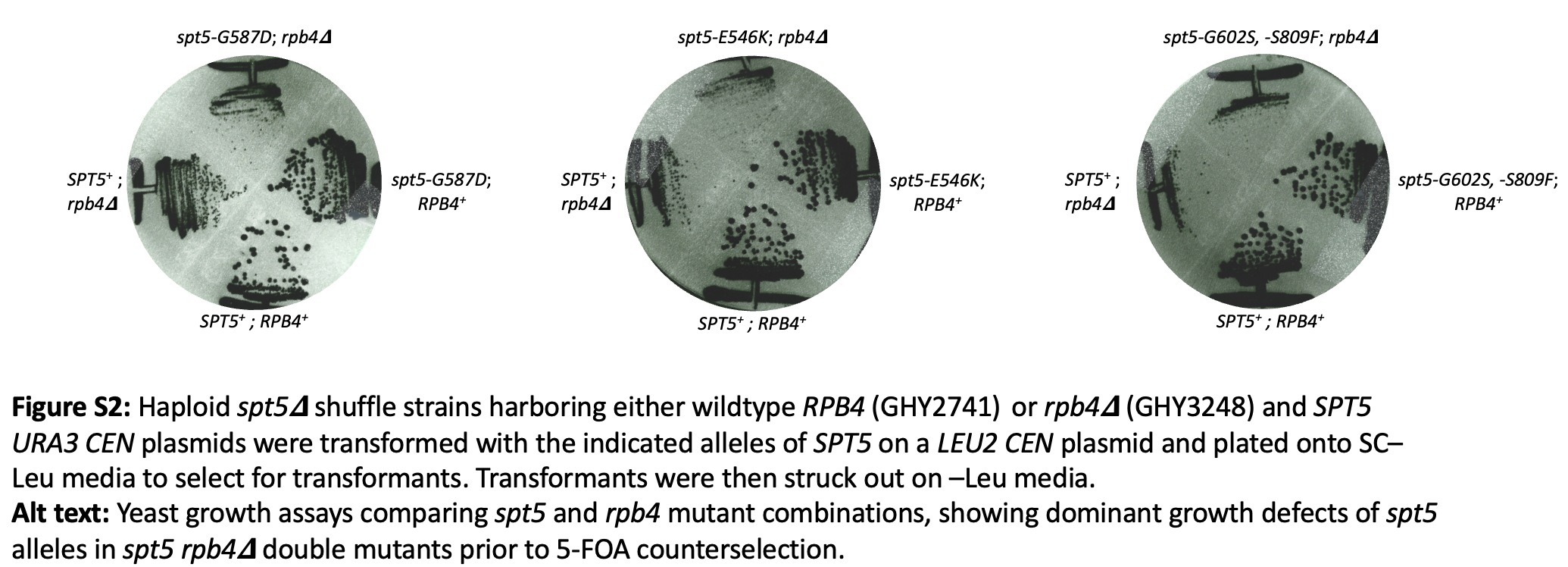

Supplement: jkag123_Supplementary_Data [file jkag123_supplementary_data.zip › Figure_S2_G3-2026-406685.tif]

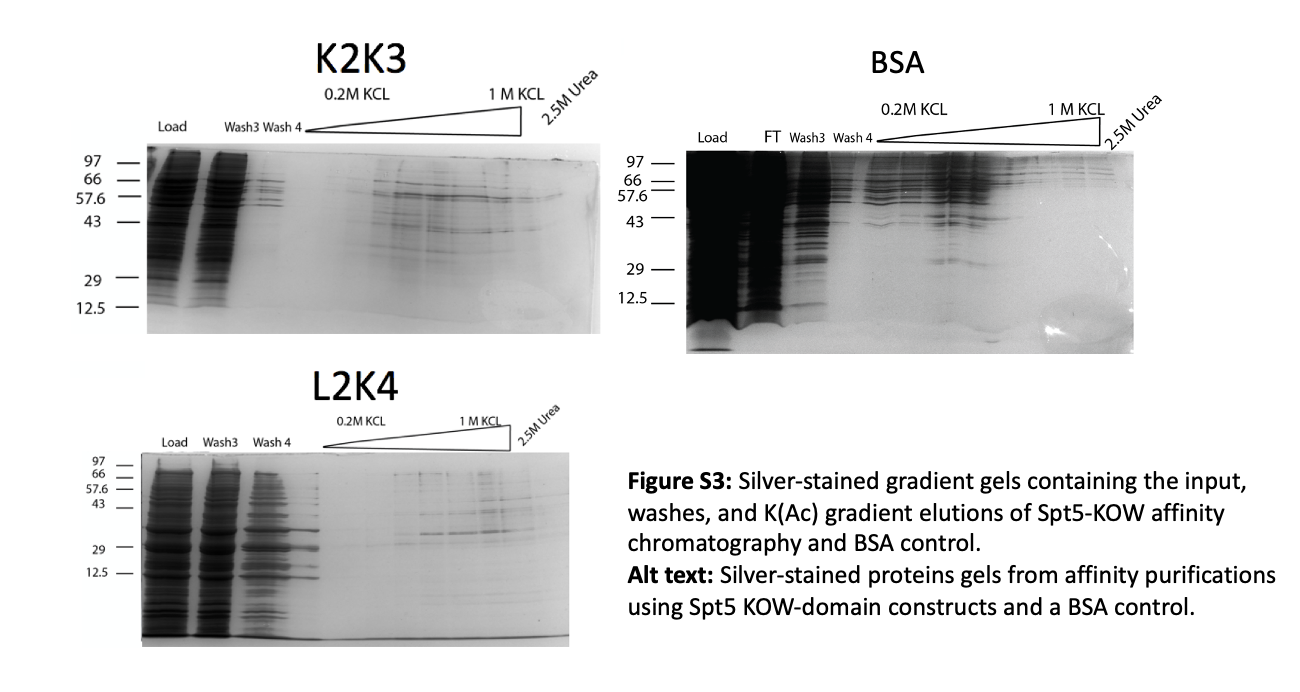

Supplement: jkag123_Supplementary_Data [file jkag123_supplementary_data.zip › Figure_S3_G3-2026-406685.tif]

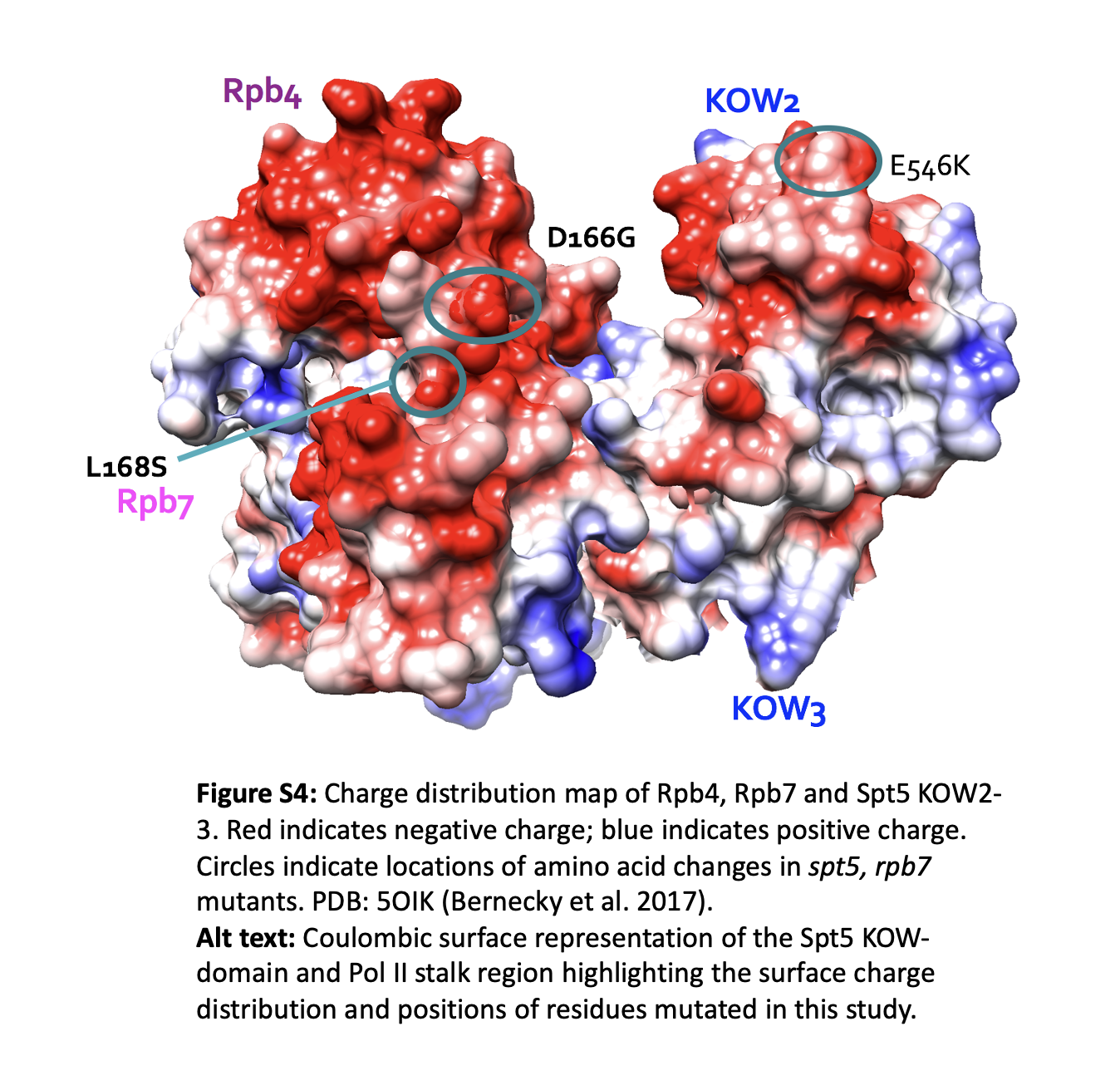

Supplement: jkag123_Supplementary_Data [file jkag123_supplementary_data.zip › Figure_S4_G3-2026-406685.tif]
